# Dicer Is Required for Normal Cerebellar Development and to Restrain Medulloblastoma Formation

**DOI:** 10.1371/journal.pone.0129642

**Published:** 2015-06-19

**Authors:** Frederique Zindy, Youngsoo Lee, Daisuke Kawauchi, Olivier Ayrault, Leila Ben Merzoug, Yang Li, Peter J. McKinnon, Martine F. Roussel

**Affiliations:** 1 Department of Tumor Cell Biology, St. Jude Children’s Research Hospital, Memphis, TN, United States of America; 2 Department of Genetics, St. Jude Children’s Research Hospital, Memphis, TN, United States of America; The University of Tennessee Health Science Center, UNITED STATES

## Abstract

Dicer, a ribonuclease III enzyme, is required for the maturation of microRNAs. To assess its role in cerebellar and medulloblastoma development, we genetically deleted Dicer in Nestin-positive neural progenitors and in mice lacking one copy for the Sonic Hedgehog receptor, *Patched 1*. We found that conditional loss of *Dicer* in mouse neural progenitors induced massive Trp53-independent apoptosis in all proliferative zones of the brain and decreased proliferation of cerebellar granule progenitors at embryonic day 15.5 leading to abnormal cerebellar development and perinatal lethality. Loss of one copy of *Dicer* significantly accelerated the formation of mouse medulloblastoma of the Sonic Hedgehog subgroup in *Patched1*-heterozygous mice. We conclude that Dicer is required for proper cerebellar development, and to restrain medulloblastoma formation.

## Introduction

MicroRNAs (miRNAs) are short non-coding RNAs derived from pri-miRNAs that are transcribed and processed by Drosha and the microprocessor complex subunit Di George syndrome critical region gene 8 (DGCR8) into pre-miRNAs in the nucleus [1,2]. Pre-miRNAs are then translocated into the cytoplasm to be further processed by Dicer into mature miRNAs. Mature single-stranded miRNAs are loaded into the RNA-induced silencing complex (RISC) that binds the 3’-UTR of mRNAs to inhibit their translation or induce their degradation [1,2]. *Dicer* is essential for early mouse development since its loss in the germline induces embryonic lethality at E7.5 [3]. However, conditional deletion of *Dicer* with Cre transgenic lines in different regions of the nervous system, including inner ear [4,5], retina [6,7], cortex and hippocampus [8–12], Purkinje cells [13], dopaminoceptive neurons [14,15], Schwann cells [16], astrocytes [17] and glia cells [18], reveals a role of Dicer and miRNAs in different developmental processes including cell survival, differentiation and proliferation. In mice, the full loss of Dicer expression is selected against in tumorigenesis [19–21], and loss of two copies of Dicer reduces cell proliferation and increases apoptosis [22]. *Dicer* is haploinsufficient for tumor suppression in some [19,20], but not all cancers [21]. In humans, germline (including *DICER1* syndrome) and somatic mutations of one copy of *DICER1* has been described in different tumor types, including one human medulloblastoma [19,23,24]. However, loss of heterozygosity is rare, but has been observed in pineoblastoma and pituitary blastoma [24].

Cerebellar granule neuron progenitors (GNPs), born in the rostral rhombic lip (rRL) at embryonic days (E) 11 to E16, migrate on the surface of the developing cerebellum to form the external granule layer (EGL) [25]. After birth, upon Sonic Hedgehog (SHH) stimulation, GNPs rapidly proliferate with maximum proliferation at postnatal days (P) 5 –P7, after which they exit the cell cycle and migrate inward to form the internal granule layer (IGL). In the mouse, the cerebellum is fully formed by 3 weeks after birth [25]. Constitutive activation of the SHH signaling pathway leads to defects in cell cycle exit, migration and differentiation which in turn can induce medulloblastoma.

The *miR-17~92* cluster family is expressed in proliferating GNPs [26] and up-regulated in SHH medulloblastoma [26–28]. Co-deletion of *miR-17~92* with its paralog *miR-106b~25* in mice reduces proliferation of GNPs at postnatal age leading to a small cerebellum [29]. While loss of *miR-17~92* leads to a relatively mild phenotype, it is absolutely required for SHH medulloblastoma formation [29]. We here show that *Dicer* that processes pre-miRNAs into mature miRNAs, including those encoded by the *miR-17~92* cluster, is required for both normal cerebellar development and medulloblastoma suppression.

## Material and Methods

### Mouse husbandry

Mouse lines carrying conditional alleles of *Dicer* (*Dicer* ^*floxed/floxed*^) [30] were generously provided by Dr. Gregory Hannon (CSHL, NY, USA). *Trp53*-null mice (stock number 002101; [31]) and the transgenic line in which the Cre recombinase is expressed under the promoter of the rat *Nestin* gene (*Nestin-Cre*) (stock number 003771; [32]) were obtained from the Jackson Laboratory (Bar Harbor, ME, USA). *Patched1*-heterozygous (*Ptch1* ^*+/-*^) mice in a *Cdkn2c-*null background (*Ptch1* ^*+/-*^; *Cdkn2c* ^*-/-*^) were previously described [33]. All mice were maintained on a mixed 129 x C57BL/6 background.

For animals that will develop medulloblastoma, we made every effort to minimize suffering by daily observation for clinical signs of sickness. Humane endpoints included dome head, titled head, loss of greater than 20% body weight, slow movements, difficulties with gait and dehydration. In the *Ptch1* ^*+/-*^; *Cdkn2c* ^*-/-*^ genetic background, ~ 60% of the mice will develop medulloblastoma while the other 40% of animals do not develop tumors. Mice with any clinical signs are not kept alive and euthanized immediately, using carbon dioxide (CO_2_).

Mice were housed in an accredited facility of the Association for Assessment and Accreditation of Laboratory Animal Care International (AAALAC). This study was carried in strict accordance with the National Institute of Health guidelines for the Care and Use of Laboratory Animals. All procedures in the protocol were approved by the Animal Care and Use Committee (ACUC) of St. Jude Children’s Research Hospital (Animal Assurance Number: A3077-01).

### Histology, immunohistochemistry and terminal deoxynucleotidyl transferase dUTP nick end labeling assay

At E14.5 and E15.5, pregnant females received intraperitoneal injections of 5-bromo-2'-deoxyuridine (BrdU at 5 mg/mL and 20 μl/g of mouse), 2 hours before euthanasia. Embryo heads were harvested, fixed overnight in 4% paraformaldehyde (PFA) in phosphate buffered saline (PBS) at 4°C, soaked in 30% sucrose in PBS until the tissues sank to the bottom of the tube, and embedded in Optimal Cutting Temperature (OCT) compound. Frozen 12 μm sections were collected on Fisherbrand superfrost plus slides using a cryostat. Slides were stained with Hematoxylin and Eosin (H&E), with antibodies to Pax6 (PRB-278P; 1/500 dilution; Covance, Emeryville, CA, USA) or BrdU (sc-32323; 1/1000 dilution; Santa Cruz, Santa Cruz, USA), as previously described [33]. Terminal deoxynucleotidyl transferase dUTP nick end labeling (TUNEL) was performed using the ApoTag Kit (EMD Millipore) following manufacturer’s instructions.

### Fluorescence in situ hybridization

Mouse embryo fibroblasts (MEF) were isolated from E14.5 *Nestin-Cre* embryos, as previously described [34]. Fluorescence In Situ hybridization (FISH) was performed as previously described [35]. In brief, metaphases were hybridized with a biotin labeled chromosome 12 BAC probe (RP23-456E21; CHORI, Oakland CA, USA) and a digoxigenin labeled probe containing the rat *Nestin* promoter. Probes were visualized by incubation with Texas red avidin and fluorescein labeled anti-digoxigenin.

### Quantitative-reverse transcriptase-polymerase chain reaction

Purification of GNPs from P7 cerebella, RNA extraction from dissected embryonic and P7 whole cerebella or from purified GNPs, and Quantitative-Reverse Transcriptase-Polymerase Chain Reaction **(**Q-RT-PCR) for *miR-19a* and *miR-106b* were performed, as previously described [26,33].

### Statistics

Statistical significance was determined using GraphPad Prism software (version 5.0). Data were shown as mean +/- s.e.m. P-values <0.05 were used as significance threshold from unpaired two-tailed Student’s *t* test. For the survival curves, p-values were determined with a log-rank (Mantel Cox) test.

## Results

### Conditional deletion of Dicer in Nestin-positive progenitor cells

**Fig 1:**
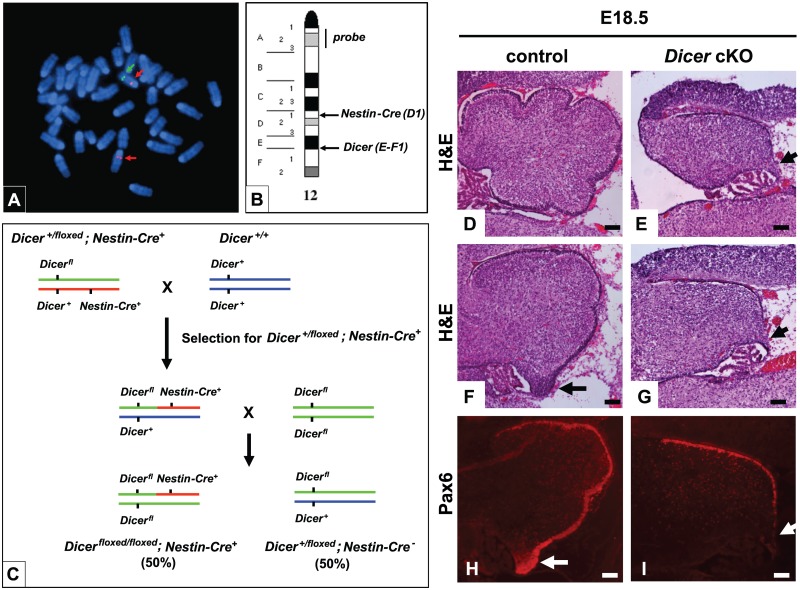
Mice lacking *Dicer* in Nestin-Cre positive cells display abnormal cerebella. (A) Chromosome spread from MEFs isolated from a *Nestin-Cre* embryo were hybridized with a biotin labeled chromosome 12 probe (red dots indicated by red arrows) and a digoxigenin labeled probe containing the rat *Nestin* promoter (green dots indicated by green arrow). The chromosomes were then stained with DAPI. A representative metaphase spread is shown. (B) Mouse chromosome 12 idiogram showing the location of chromosome 12 probe, *Dicer* and *Nestin-Cre* genes. (C) *Dicer* ^*floxed/+*^ *; Nestin-Cre* ^*+*^ were crossed with C57BL/6 mice to select for *Dicer* ^*floxed/+*^ *; Nestin-Cre* ^*+*^ in which *Dicer* and *Nestin-Cre* were located on the same chromosome and subsequently crossed with *Dicer* ^*floxed/floxed*^ *; Nestin-Cre* ^*-*^ to obtain *Dicer* ^*floxed/floxed*^ *; Nestin-Cre* ^*+*^ (referred as *Dicer* cKO) and *Dicer* ^*floxed/+*^ *; Nestin-Cre* ^-^ (referred as control). (fl) floxed allele for *Dicer*. (D, E) Representative mid-sagittal and (F-I) para-sagittal sections through the E18.5 developing cerebellum of control (D, F, H) and *Dicer* cKO (E, G, I) mice. Tissues stained with H&E (D-G) or a Pax6 antibody (H, I, red). Arrows indicate the rostral rhombic lip. (n = 2 independent embryos; scale bars = 100μm).

To assess the role of mature microRNAs in brain development, we crossed conditional *Dicer-*floxed mice with the *Nestin-Cre* transgenic line and generated *Dicer* ^*floxed/floxed*^; *Nestin-Cre* ^*+*^ mice (referred as *Dicer* cKO). We found that the *Nestin-Cre* transgene was located on chromosome 12 (band D1) and genetically linked to *Dicer* (band E-F1) by ~25 megabases (Fig 1A and 1B). Thus, the generation of *Dicer* cKO animals required that the *Nestin-Cre* transgene and one floxed allele of *Dicer* be carried by the same chromosome which necessitated a complex breeding strategy (Fig 1C). *Dicer* cKO mice died at birth (15/15) but were alive at E18.5. When compared to control littermates (Fig 1D, 1F and 1H), cerebella from E18.5 *Dicer* cKO embryos had a reduced size and lacked foliations (Fig 1E, 1G and 1I), a thinner EGL visualized with an antibody to Pax6 (Fig 1I) and the rRL devoid of Pax6-expressing progenitor neurons (Fig 1I, white arrows). In contrast, the cerebellum of *Dicer* ^*floxed/+*^; *Nestin-Cre* ^*+*^ mice appeared normal (data not shown).

**Fig 2:**
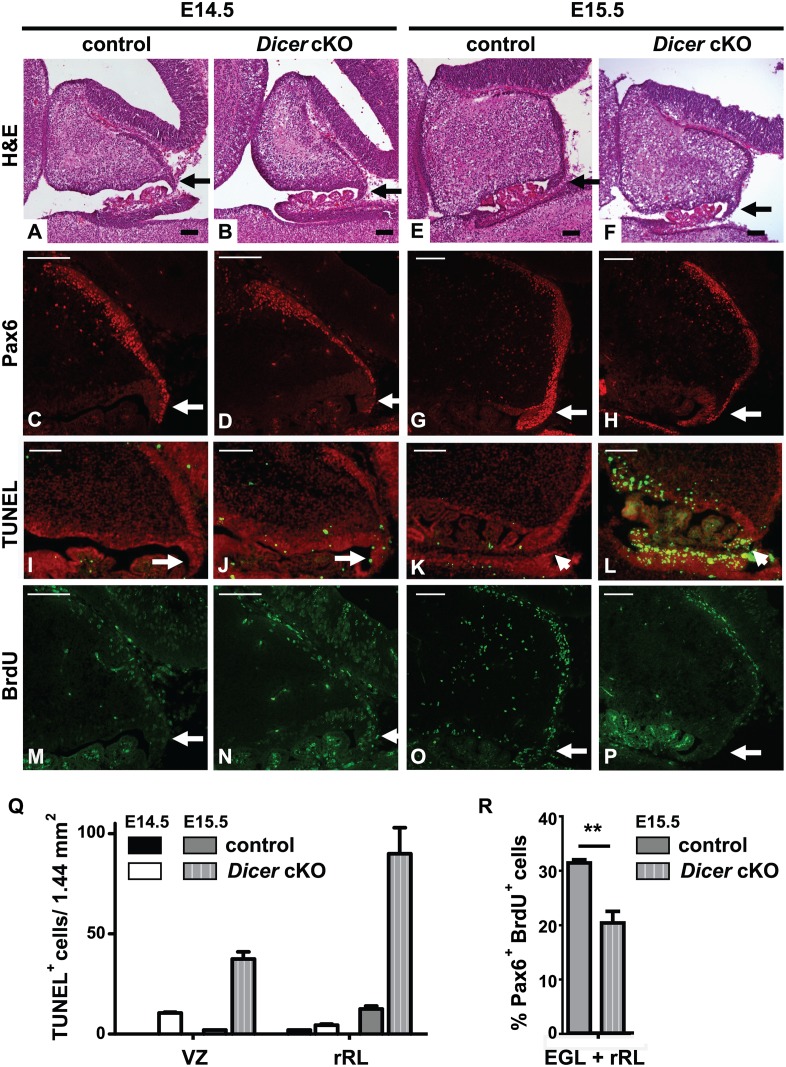
Conditional loss of *Dicer* induces apoptosis of neural progenitors from the rostral rhombic lip. Representative sagittal sections through the developing cerebellum of control littermates and *Dicer* cKO mice at E14.5 (A-D, I, J) and E15.5 (E-H, K, L) stained with H&E (A, B, E, F), an antibody to Pax6 (C, D, G, H, red), TUNEL (I-L green), and a BrdU antibody (M-P, green). Nuclei were counterstained with propidium iodide (I-L, red). Black and white arrows indicate the rRL. E14.5 n = 2, E15.5 n = 3, independent embryos. Black and white scale bars = 100μm. (Q) Number of TUNEL^+^ cells were counted on a 1.44mm^2^ surface within the ventricular zone (VZ) and the rostral rhombic lip (rRL) from 2 independent brain sections of one embryo per genotype. (R) Percentage of Pax6^+^, BrdU^+^ cells in the rRL and the external granule layer (EGL) of n = 3 control and n = 3 *Dicer* cKO mice. (**) p-value = 0.0073.

To identify the cause of abnormal cerebellar development, we analyzed embryos at earlier time points. At E14.5, the rRL and the nascent EGL of the *Dicer* cKO mice appeared similar to control littermates (Fig 2A and 2B). Pax6 positive cells (Pax 6^+^) were similarly present in the exterior face of the rRL and in the nascent EGL of both *Dicer* cKO and control mice (Fig 2C and 2D). In contrast, at E15.5, the nascent EGL of the *Dicer* cKO mice were smaller than in control animals (Fig 2E and 2F), and the exterior face of the rRL appeared thin (Fig 2G and 2H, white arrows). TUNEL staining revealed few apoptotic cells at E14.5 (Fig 2I, 2J and 2Q). In contrast, massive apoptosis was observed in the rRL but not in the EGL, at E15.5 (Fig 2K, 2L and 2Q). In *Dicer* cKO mice at E15.5 by *in vivo* BrdU labeling we observed decreased proliferation of the Pax6^+^ cells in the rRL and EGL (Fig 2P and 2R). Because late-born GNPs contribute to the formation of the posterior EGL [36], the loss of Pax6^+^ cells in *Dicer*-deficient posterior cerebella at E18.5 could be explained by the apoptosis of cells in the rRL and their reduced proliferation in the rRL and EGL at E15.5. In addition to the rRL, the *Nestin-cre* transgene is widely expressed in proliferative zones throughout the central nervous system (CNS). Consistent with this expression pattern, massive apoptosis was observed in the cerebellar ventricular zone (VZ) and caudal rhombic lip (cRL) (Fig 2L and 2Q).

**Fig 3:**
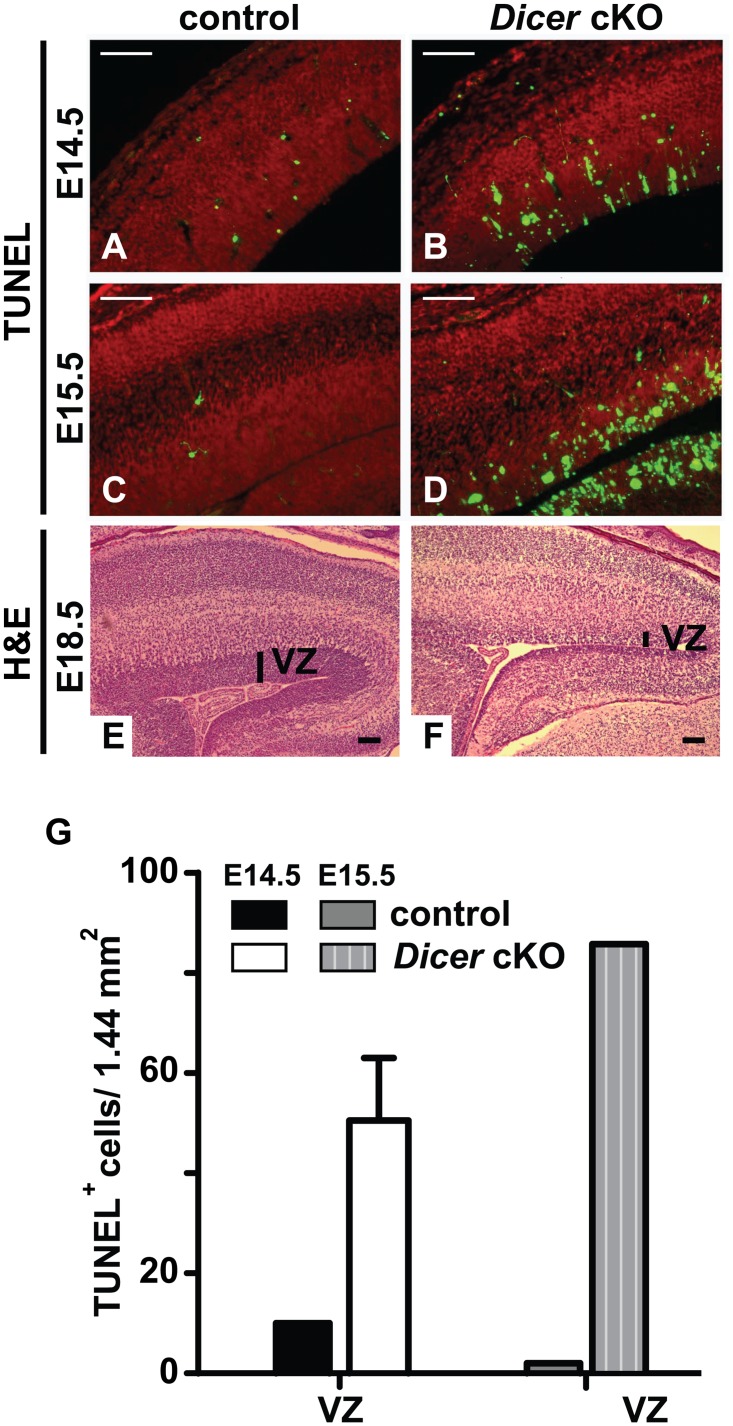
Massive apoptosis in the cerebral cortex of the *Dicer* cKO embryos. Representative TUNEL staining (green) was performed on sagittal sections of the cortex (A-D) from a control (A, C) and *Dicer* cKO (B, D) mouse at E14.5 (A, B) and E15.5 (C, D). Nuclei were counterstained with propidium iodide (red). Representative sagittal sections through the cortex of an E18.5 control (E) and *Dicer* cKO (F) mouse were stained with H&E. Vertical black bars indicate the ventricular zone (VZ). n = 2 independent embryos. Black and white scale bars = 100μm. (G) TUNEL^+^ cells were quantified in the VZ from 2 independent brain sections of one embryo per genotype.

Apoptosis was already detected in the neocortex of *Dicer* cKO embryos from E14.5 onward (Fig 3A–3D and 3G), as previously reported [10,12] and in the VZ of the colliculus (data not shown). The VZ of the neocortex generates neurons that radially migrate towards the surface of the brain to their final position with a specific address dependent on their birth date resulting in a cortex with characteristic six neuronal layers (Fig 3E). In the *Dicer* cKO mice, at E18.5, the cortex was less laminated with a thin VZ (Fig 3F).

**Fig 4:**
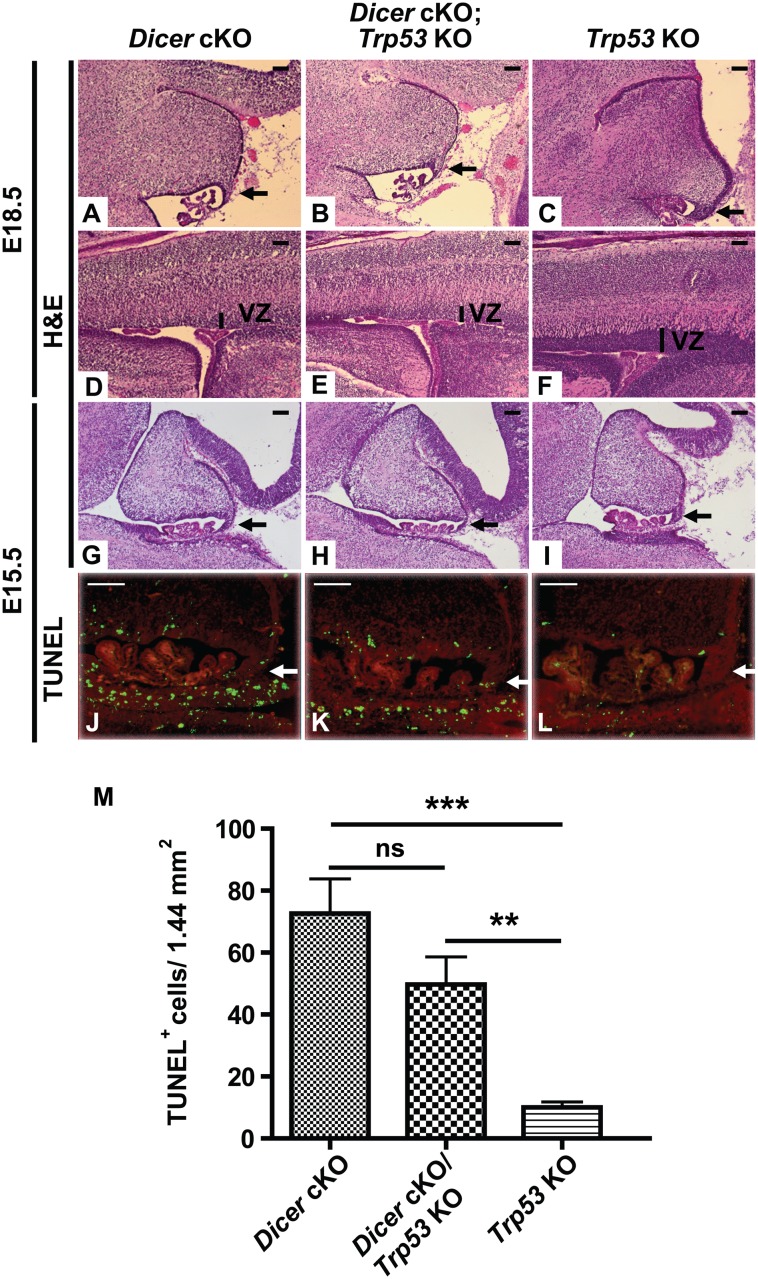
Deletion of *Trp53* is unable to rescue the phenotypes observed in *Dicer* cKO mice. Representative sagittal sections through the developing cerebellum (A-C, G-L) and cortex (D-F) of *Dicer* cKO (A, D, G, J), *Dicer* cKO; *Trp53* KO (B, E, H, K, and *Trp53* KO (C, F, I, L) mice at E18.5 (A-F) and E15.5 (G-L) were stained with H&E (A-I) and by TUNEL (J-L). Nuclei were counterstained with propidium iodide (red) (J-L). Black and white arrows indicate the rostral rhombic lip. Vertical black bars indicate the ventricular zone (VZ). (n = 2 independent E18.5 or 3 E15.5 embryos. Scale bars = 100μm). (M) TUNEL^+^ cells were counted in the VZ and rRL from 2 independent brain sections of 3 embryos per genotypes. (***) p-values = 0.0002; (**) p-values = 0.0014 and ns (non-significant)

Apoptosis can proceed by a Trp53-dependent pathway, particularly in the case of induced genome instability, or by Trp53-independent pathways [37, 38]. To determine if the cell death resulting from Dicer loss was a result of DNA damage, we asked if the apoptosis was dependent on Trp53 function. Loss of *Trp53* did not rescue lethality, nor the phenotypes observed in the developing brain of the *Dicer* cKO mice (i.e. apoptosis, abnormal cerebellum and less laminated cortex) (Fig 4A–4M), suggesting that apoptosis and other phenotypes did not result from DNA damage.

### MicroRNAs are progressively deleted in the cerebellum of Dicer cKO embryos

**Fig 5:**
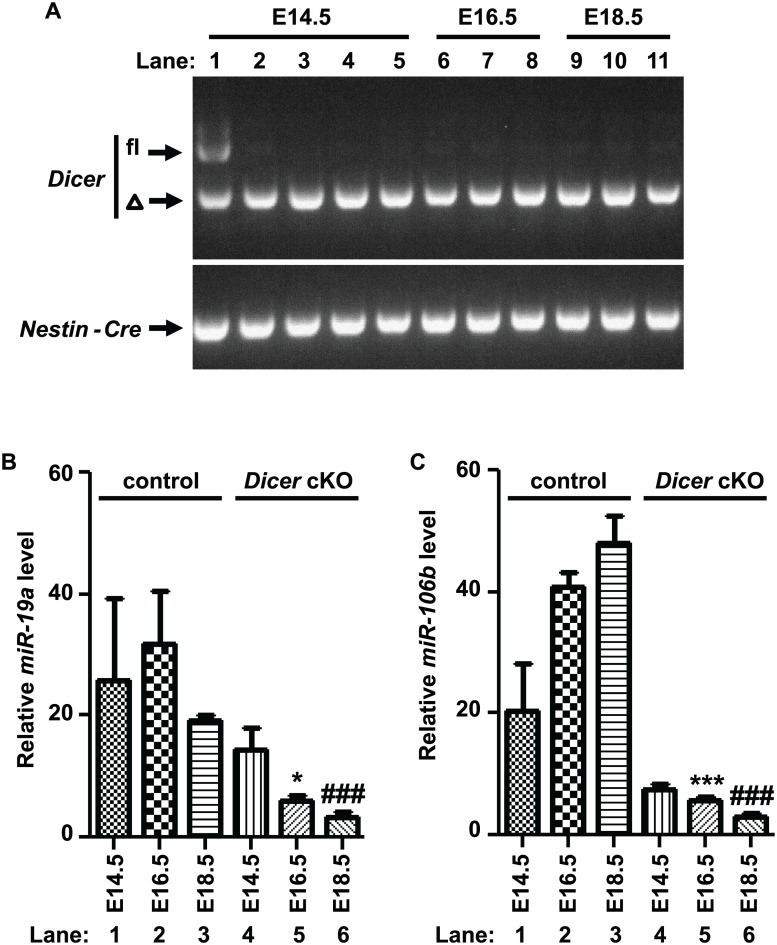
Delayed loss of microRNAs in *Dicer* cKO embryos. (A) PCR for *Dicer* and *Nestin-Cre* was performed on DNA extracted from tail (lane 1) or total cerebella (lanes 2–11) of *Dicer* cKO mice at E14.5 (lanes 1–5), E16.5 (lanes 6–8) and E18.5 (lanes 9–11); *Dicer* alleles: (fl) floxed, (Δ) deleted. Relative levels of mature microRNAs *miR-19a* (B) and *miR-106b* (C) were determined by Q-RT-PCR on total RNA extracted from total cerebella of control (lanes 1–3) and *Dicer* cKO (lanes 4–6) mice at E14.5 (lanes 1, 4), E16.5 (lanes 2, 5) and E18.5 (lanes 3, 6). (*) p-values <0.05, (***, ###) p-values <0.001. n = 3 independent samples. (*) p-values were calculated by comparing lane 2 to lane 5 and (#) lane 3 to lane 6.

Cre recombinase activity under the control of the *Nestin* promoter is initiated between E9.5 and E10.5 [39]. However, we observed massive apoptosis at E15.5 in the rRL and the VZ of the developing cerebella in *Dicer* cKO mice, suggesting that complete loss of Dicer and therefore of mature microRNAs may have happened progressively from E9.5 to E15.5. We confirmed that the two *Dicer* alleles were deleted in the cerebellum at E14.5 (Fig 5A). We previously reported that the *miR-17~92* cluster family is expressed in the developing cerebellum [29]. To evaluate how the loss of Dicer affects the expression of mature microRNAs, we determined the expression of *miR-19a* (from the *miR-17~92* cluster) and *miR-106b* (from the *miR-106b~25* cluster) in the developing cerebella of control and *Dicer* cKO embryos at E14.5, E16.5 and E18.5 by Q-RT-PCR (Fig 5B and 5C, lanes 1–6). While the levels of *miR-19a* and *miR-106b* were not statistically different at E14.5 (Fig 5B and 5C, lane 1 versus lane 4), their levels were significantly reduced at E16.5 (Fig 5B and 5C, lane 2 versus lane 5, p = 0.0386 and 0.0001, respectively) and at E18.5 (Fig 5B and 5C, lane 3 versus lane 6, p = 0.0005 and 0.0005, respectively). Therefore deletion of *Dicer* resulted in a delayed decrease of *miR-19a* and *miR-106b* microRNAs with significant loss of those microRNAs at E16.5.

### Dicer restrains medulloblastoma development

**Fig 6:**
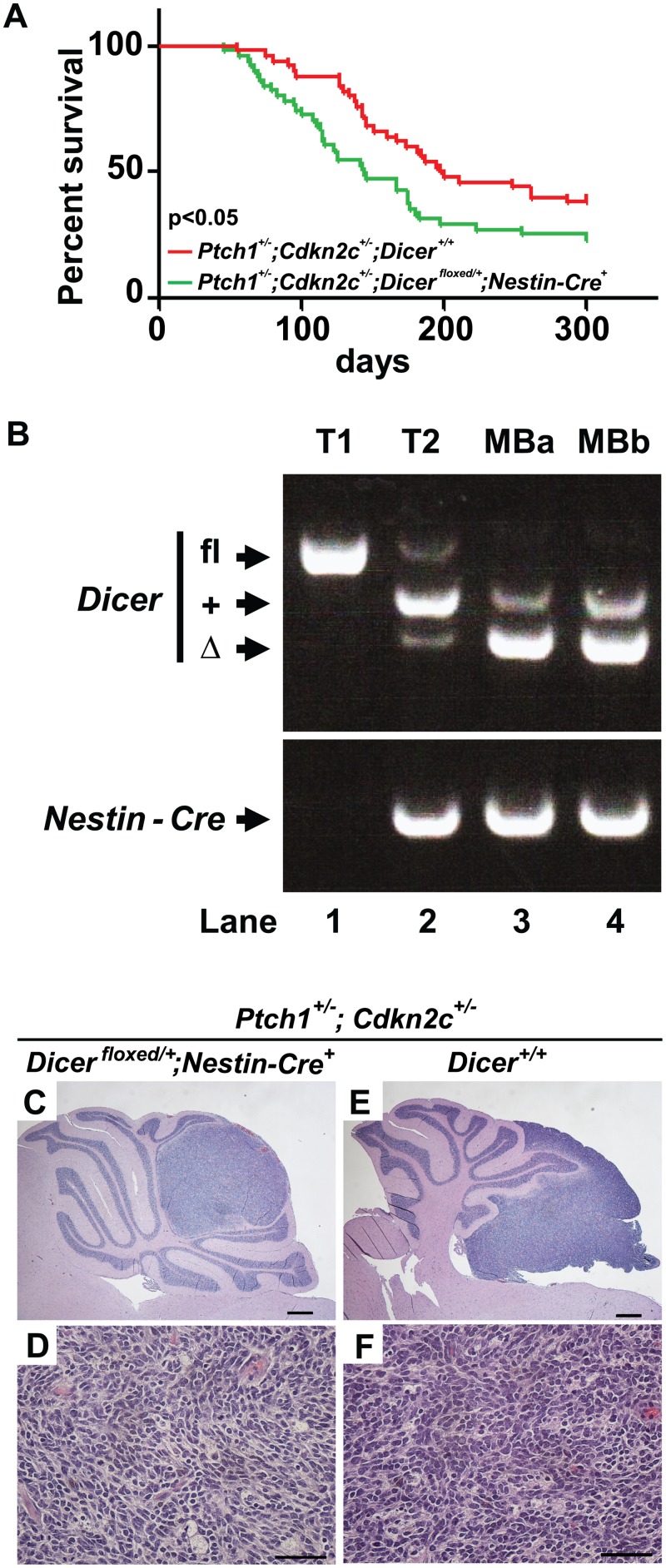
*Dicer* restrains medulloblastoma development. (A) Survival curves for *Ptch1* ^*+/-*^; *Cdkn2c* ^*+/-*^ mice with different *Dicer* and *Nestin-Cre* genotypes. *Dicer* ^*floxed/+*^; *Nestin-Cre* ^*+*^ *;* (green line, n = 51) and *Dicer* ^+/+^ (red line, n = 50). (B) PCR for *Dicer* and *Nestin-Cre* was performed on DNA extracted from tail (T1) of a *Dicer* ^*floxed/floxed*^; *Nestin-Cre* ^*-*^; *Ptch1* ^*+/-*^; *Cdkn2c* ^*+/-*^ mouse, tail (T2) and two independent pieces of the same medulloblastoma (MBa and MBb) from a *Dicer* ^*floxed/+*^; *Nestin-Cre* ^*+*^; *Ptch1* ^*+/-*^; *Cdkn2c* ^*+/-*^ mouse. *Dicer* alleles: (fl) floxed, (+) wild type, (Δ) deleted. (C-F) Representative H&E staining of medulloblastoma in (C) *Dicer* ^*floxed/+*^; *Nestin-Cre* ^*+*^; *Ptch1* ^*+/-*^; *Cdkn2c* ^*+/-*^ and (E) *Dicer* ^*+/+*^; *Ptch1* ^*+/-*^; *Cdkn2c* ^*+/-*^ mice. Scale bars = 500μm (C and E) and 50μm (D and F).

*Dicer* was previously reported to be haploinsufficient for tumor suppression in several mouse and human cancers [19,20]. SHH medulloblastoma arise from GNPs with aberrant SHH signaling upon deletion of one copy of the SHH receptor Patched (*Ptch1* ^+/-^) alone or with one or two copies of *Cdkn2c* [33]. To determine if Dicer was haploinsufficient for SHH medulloblastoma development, *Dicer* ^*floxed/+*^; *Nestin-Cre* ^*+*^ animals were bred to the *Ptch1* ^*+/-*^; *Cdkn2c* ^*-/-*^ mice. Loss of one copy of *Dicer* induced SHH medulloblastoma with a similar time of onset and penetrance in *Dicer* ^*floxed/+*^; *Nestin-Cre* ^*+*^; *Ptch1* ^*+/-*^; *Cdkn2c* ^*+/-*^ compared to *Dicer* ^*+/+*^; *Ptch1* ^*+/-*^; *Cdkn2c* ^*+/-*^ mice, 76.5% (39/51) versus 62% (31/50), respectively (Fig 6A). In contrast, *Dicer* ^*floxed/+*^; *Nestin-Cre* ^*+*^; *Ptch1* ^*+/-*^; *Cdkn2c* ^*+/-*^ mice died significantly faster (median survival, 144 days) than *Dicer* ^*+/+*^; *Ptch1* ^*+/-*^; *Cdkn2c* ^*+/-*^ mice (198.5 days) (p = 0.0247) (Fig 6A). We confirmed by PCR that the floxed allele but not the wild type allele of *Dicer* was deleted (Fig 6B, lanes 1–4). Analysis of tumors pathology from the two genotypes did not reveal differences and confirmed that they were medulloblastoma of the SHH subgroup with classic morphology of round cells (Fig 6C–6F). These results suggest that *Dicer* restrains SHH medulloblastoma development and is haploinsufficient for tumor suppression.

## Discussion

We here report the role of Dicer and mature miRNAs during cerebellar development and SHH medulloblastoma genesis by conditional deletion of *Dicer* (*Dicer* cKO) in Nestin-positive neural progenitors. We found that conditional inactivation of *Dicer* triggered extensive *Trp53*-independent apoptosis in all proliferative zones throughout the developing CNS and decreased proliferation of GNPs in the developing cerebellum. Loss of one copy of *Dicer* accelerated medulloblastoma formation in *Ptch1* ^*+/-*^, *Cdkn2c* ^*+/-*^ animals. *Dicer* was shown by several groups to be required for proper development of many neural cell populations in the developing CNS [8–14,18]. Conditional loss of *Dicer* in the CNS was previously found to induce apoptosis using the same [10,12] or different [8,9,11,13,17,18,40] *Cre* lines, and to affect cortical development [8–10,12]. While using the same *Nestin*-*Cre* and *Dicer*-floxed lines, s, the group of Kawasa-Koga did not find obvious defects cerebellar anomalies in their *Dicer* cKO mice [10]. They show that while miRNAs are still detected at E15.5 in the cortex of their animals, they are absent at E18.5 correlating with apoptosis. Kawasa-Koga and colleagues remarked that *Nestin-Cre; Dicer* mice produced very few *Dicer* conditional knockout mice embyos, 6.8% at E18.5 and that no surviving newborns were detected. This is consistent with our data and the finding that *Dicer* and *Nestin*-*Cre* are genetically linked on mouse chromosome 12. In the Discussion of their paper, Kawase-Koga and colleagues did not find obvious defects in cerebellar development during embryonic stages and yet, they obtained very few and no live embryos while detecting defects in the cortex of *Nestin-Cre; Dicer* mice. These differences in phenotypes between groups may potentially be due to the genetic background. Using the same *Nestin*-*Cre* but different *Dicer*-floxed mice, McLoughlin and collaborators also describe cortical defects in *Dicer* cKO mice, but did not report on the status of the mice cerebella [12]. McLoughlin and colleagues used *Dicer-floxed* animals generated by Harfe and colleagues. In their manuscript, they only described the effect of Dicer deletion in the cortex but not in the cerebellum and found increased apoptosis in cortical regions at E15.5, which is consistent with our data. They also detected low numbers of *Dicer* conditional knockout mice embryos and no surviving embryos. Kawase-Koga and McLoughlin focused on cortical development while we assessed the role of Dicer in the embryonic cerebellum. To increase the percentage of embryos with the proper genotype, we backcrossed our mice into C57BL/6 background (Fig 1C) that changed the genetic background of our cohort, compared to that of Kawase-Koga and colleagues. This may explain why Kawase-Koga and colleagues do not see apoptosis at E15.5 in the cortices of *Nestin-Cre* ^*+*^; *Dicer* ^*floxedl/floxed*^ mice; even though they show, by immunoblotting, that the levels of Dicer protein are greatly reduced at E15.5. They also note that Dicer deletion occurs at different times depending on the localization.

Despite these few differences, our results are consistent with these reports since conditional deletion of *Dicer* in Nestin-positive cells induced substantial apoptosis in all proliferative zones of the CNS.

In *Dicer* cKO mice, loss of neural progenitors in the rRL led to a diminished pool of GNPs which resulted in an abnormally developed cerebellum with a short and thin EGL, absence of folia and an aberrant residual rRL at E18.5. This was associated with apoptosis in the rRL and reduced proliferation of GNPs in the developing EGL. Apoptosis was also observed in the ventricular zone of the developing cerebellum. These results suggest that lack of Dicer induced apoptosis in stem / progenitor neural cells, before they commit to their final lineages and controlled the proliferation of GNPs in the developing EGL

Although *Nestin* is expressed as early as E9.5–10.5 in neural progenitors [39], the decrease in microRNAs levels in the developing cerebella of the *Dicer* cKO mice was delayed, consistent with the relatively long half-life of miRNAs [41]. At E14.5, the cerebella of *Dicer* cKO and control littermates were normal and the levels of miRs were not significantly reduced. However, at E15.5, massive apoptosis was observed, suggesting that residual levels of microRNAs were insufficient to maintain the proliferation of neural progenitors. Overall, our results suggest that processing of microRNAs by Dicer was required for proper cerebellar development during embryogenesis. Alternatively, the effects observed might be due to miR-independent cell survival function of Dicer, as previously reported [42]. Thus, our study is the first analysis of the role of Dicer during cerebellar development. We consider that our data and that from others illustrate a critical role for this protein, in the regulation of *miRNAs* during development of the cerebellum.

We found that *Dicer* restricts SHH medulloblastoma development in *Ptch1* ^*+/-*^ mice. Our results are consistent with those reported in two mouse models of retinoblastoma and lung cancers [19,20] but not in Eμ-Myc B cell lymphoma [21]. We previously showed that *miR-17~92* expression is absolutely required for SHH medulloblastoma initiation [29] and that its silencing inhibits medulloblastoma progression [43]. This implies that complete loss of Dicer is selected against medulloblastoma formation and progression. Here we show that loss of one copy of *Dicer* in a SHH medulloblastoma mouse model accelerated tumor formation, suggesting that decreased levels of some mature tumor suppressor *miRNAs* might promote tumorigenesis. We will unable to correlate the phenotypes of *Dicer* cKO in embryos with the ones we observed with the *mir17~92* cluster family because the effects of the loss of the *mir17~92* cluster family are only revealed in the cerebellum after birth [29]. The *Dicer* cKO mice instead die at birth. We previously published that the *miR-17~92* cluster family was required for proliferation of GNPs during postnatal cerebellar and medulloblastoma development [29,43]. In the present study, we used two of the microRNAs encoded by the *miR-17~92* cluster family (*miR-19a* and *miR106b*) to determine the level of microRNA biogenesis. In previous studies, we determined that these two miRNAs were expressed during embryonic cerebellar development [29]. Clearly other microRNAs besides those encoded by the *miR-17~92* cluster family are expressed during cerebellar development. Therefore, the apoptosis observed at E15.5 *Dicer* cKO embryos is most likely due, only in part, to the loss of expression of microRNAs encoded by the *miR-17~92* cluster family.

In summary, while *Dicer* is essential for proper cerebellar development and homeostasis, it restrains SHH medulloblastoma development suggesting that it might be haploinsufficient for medulloblastoma suppression.
